# MAPK Phosphatase-3 Mediates Chronic Endoplasmic Reticulum Stress Promoting Hepatic Gluconeogenesis

**DOI:** 10.3390/ijms27062874

**Published:** 2026-03-22

**Authors:** Sheng Cao, Yanlin Du, Zhengfeng Fang, Lianqiang Che, Yan Lin, Shengyu Xu, Xuemei Jiang, Guangmang Liu, Yong Zhuo, Lun Hua, Mengmeng Sun, De Wu, Bin Feng

**Affiliations:** 1 Animal Nutrition Institute, Sichuan Agricultural University, 211 Huimin Road, Wenjiang District, Chengdu 611130, China; cao420827@163.com (S.C.); 15680557909@163.com (Y.D.); zfang@sicau.edu.cn (Z.F.); clianqiang@hotmail.com (L.C.); linyan@sicau.edu.cn (Y.L.); shengyu_x@hotmail.com (S.X.); 71310@sicau.edu.cn (X.J.); liugm@sicau.edu.cn (G.L.); zhuoyong@sicau.edu.cn (Y.Z.); hualun@sicau.edu.cn (L.H.); wude@sicau.edu.cn (D.W.); 2 Key Laboratory of Animal Disease-Resistant Nutrition of Ministry of Education, Sichuan Agricultural University, 211 Huimin Road, Wenjiang District, Chengdu 611130, China; 3 Animal Nutrition and Feed Efficient Utilization Key Laboratory of Sichuan Province, Sichuan Agricultural University, 211 Huimin Road, Wenjiang District, Chengdu 611130, China; 4 College of Food Science, Sichuan Agricultural University, Ya’an 625014, China; 5 College of Science, Sichuan Agricultural University, Ya’an 625014, China; 14391@sicau.edu.cn

**Keywords:** ER stress, PERK, MKP-3, gluconeogenesis, liver

## Abstract

Long-term nutritional excess causes hepatic steatosis, endoplasmic reticulum (ER) stress, hyperglycemia, and hyperlipidemia. Mitogen-activated protein kinase phosphatase-3 (MKP-3) is a well-established stress-regulated protein and a regulator of gluconeogenesis. Our previous study revealed that acute ER stress reduced gluconeogenesis and MKP-3 protein stability. However, the expression of MKP-3 and its regulatory mechanisms in chronic ER stress remain unclear. The aim of this study was to investigate the effects of chronic ER stress on hepatic MKP-3 expression and its role in the regulation of gluconeogenesis. The results show that long-term administration of thapsigargin (Tg) or palmitic acid promoted gene expression of *Mkp-3* and gluconeogenic genes *Pepck*, *G6pc*, and *Pgc1α* in primary mouse hepatocytes. In addition, a long-term high-fat diet (HFD) or Tg administration significantly increased hepatic ER stress and blood glucose level in mice, while inducing the expression of *Mkp-3* and hepatic gluconeogenic genes *Pepck*, *G6pc* and *Pgc1α*. Further study revealed that liver-specific *Mkp-3* knockout (*Mkp-3* LKO) reversed the blood glucose level and expression levels of gluconeogenic genes those were induced by long-term HFD in mice. Moreover, activation of the PKR-like ER kinase (PERK) by its agonist increased hepatic *Mkp-3* expression, whereas inhibitor of PERK suppressed the expression of *Mkp-3* under Tg administration. These results suggest that chronic high-fat diet might promote hepatic gluconeogenesis via the PERK/MKP-3 pathway. Consequently, this study identified a potential therapeutic target for treating obesity-related hyperglycemia.

## 1. Introduction

Obesity-related hyperglycemia has emerged as a significant global health concern, contributing to the increasing incidence of chronic diseases such as type 2 diabetes mellitus (T2DM) and cardiovascular disease. The pathogenesis of these disorders is complex and multifaceted, involving dietary factors and a variety of cellular responses [1]. Endoplasmic reticulum (ER) stress is a key cellular stress response that has garnered substantial research attention in recent years, with studies demonstrating its close association with metabolic diseases [2].

The liver plays a key role in glucose and lipids metabolism. Under normal conditions, hepatic gluconeogenesis is activated by glucagon when the body is in a fasting or starvation state, and is inhibited by insulin when the body is in a feeding state. The liver maintains normal blood glucose levels during fasting or starvation primarily through gluconeogenesis. However, prolonged nutritional excess (such as a high-energy, high-fat or high-protein diet) can lead to disorders of lipid metabolism, accumulation of misfolded proteins and disruption of calcium homeostasis. Multiple pathways, including the activation of oxidative stress and inflammatory signaling, induce chronic ER stress in cells. In the liver in particular, persistent chronic ER stress disrupts normal hepatocyte metabolic processes, leading to multiple metabolic disorders. This increases hepatic gluconeogenesis, which triggers hyperglycemia, insulin resistance and hepatic dysfunction [3,4,5,6].

The ER plays a central role in maintaining cellular homeostasis, particularly during the synthesis, folding, and secretion of proteins. Chronic ER stress is activated when the ER becomes overburdened or when unfolded proteins accumulate significantly. This triggers the unfolded protein response (UPR), which aims to restore normal ER function. ER stress can be induced by numerous factors, and different types of ER stress mediate distinct signaling pathways [7,8]. ER stress can be categorized into three types based on its triggers and duration: acute ER stress (induced by chemicals), chronic ER stress (caused by a steady-state imbalance), and physiological ER stress (caused by metabolic fluctuations). Physiological ER stress primarily arises from fasting, hunger cycles, or other rhythmic life activities and is a normal physiological phenomenon [9]. Acute ER stress is chiefly triggered by short-term exposure to chemical molecules, such as saturated fatty acids, cholesterol, mycotoxins (e.g., ergotamine), and toxic carotenoids. This induces acute metabolic disruption and even cytotoxicity. Chronic ER stress is primarily induced by prolonged nutritional excess [10]. Three classical ER stress signaling pathways are currently recognized: the protein kinase R-like ER kinase (PERK) pathway, the inositol-dependent enzyme 1 (IRE1) pathway, and the activating transcription factor 6 (ATF6) pathway [11]. Of these pathways, the PERK pathway is identified as the regulator of glucose and lipids metabolism [12,13].

Mitogen-activated protein kinase phosphatase-3 (MKP-3) has been reported to stimulate gluconeogenesis, and can be regulated by insulin, glucocorticoid and leptin [14,15,16]. Previous studies have demonstrated that, in the absence of the MKP-3 gene, both lean and obese mice exhibit reduced blood glucose concentrations during fasting [16,17]. Conversely, overexpression of MKP-3 promotes hepatic gluconeogenesis. Our previous study has shown that MKP-3 positively regulates hepatic gluconeogenesis by dephosphorylating forkhead box O1 (FOXO1) and increasing peroxisome proliferator-activated receptor gamma coactivator 1 alpha (PGC-1α) expression [17]. Furthermore, acute ER stress inhibits hepatic gluconeogenesis by promoting MKP-3 degradation, indicating MKP-3’s pivotal role in glucose metabolism [18]. However, the role of MKP-3 in chronic ER stress-mediated gluconeogenesis remains to be investigated. The current study analyzed the effects of chronic ER stress induced by excess energy intake or a chemical on hepatic gluconeogenesis and *Mkp-3* expression level in both pigs and mice. In addition, the molecular mechanisms by which chronic ER stress regulates *Mkp-3* expression has also been investigated.

## 2. Results

### 2.1. Chronic ER Stress Promotes Gluconeogenesis and MKP-3 Expression in Primary Mouse Hepatocytes

**Figure 1:**
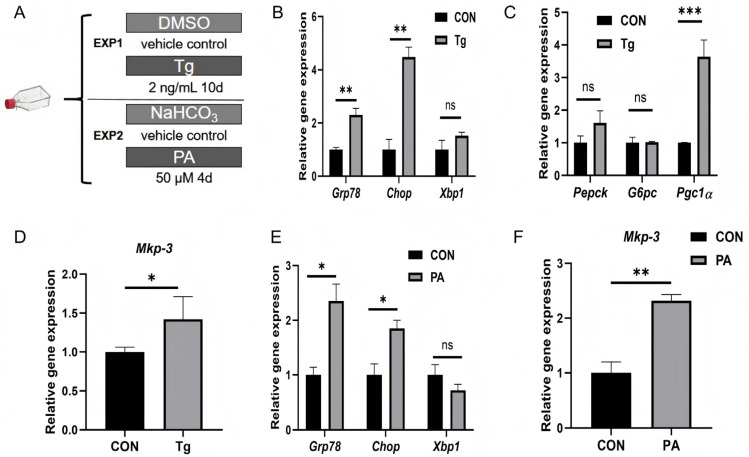
Chronic ER stress promoted gluconeogenesis and *Mkp-3* expression. (**A**) Primary mouse hepatocytes were treated with Tg (2 ng/mL) for 10 days or cultured with PA (50 µM) for 4 days, or with their respective controls. (**B**) Expression levels of ER stress marker genes *Grp78*, *Chop* and *Xbp1* in Tg-treated hepatocytes (*n* = 3). (**C**) Expression levels of gluconeogenic genes *Pepck* and *G6pc*, and their regulator *Pgc1α* in Tg-treated hepatocytes (*n* = 3). (**D**) Expression level of *Mkp-3* in Tg-treated hepatocytes (*n* = 3). (**E**) Expression of ER stress marker genes *Grp78*, *Chop* and *Xbp1* in PA-treated hepatocytes (*n* = 3). (**F**) Expression level of *Mkp-3* in PA-treated hepatocytes (*n* = 3). Data are presented as mean ± standard error. * *p* < 0.05, ** *p* < 0.01, *** *p* < 0.001, ns indicates no significant difference.

To investigate the in vitro effects of chronic ER stress mediated by chemicals on gluconeogenesis, primary mouse hepatocytes were treated with thapsigargin (Tg, 2 ng/mL) for 10 days (Figure 1A). Results showed that Tg significantly increased the expression of ER stress marker genes *Grp78* and *Chop* compared to control (Figure 1B). In addition, the gluconeogenic gene *Pgc1α* was significantly upregulated by Tg, while phosphoenolpyruvate carboxykinase (*Pepck*) or glucose-6-phosphatase (*G6pc*) remained unchanged (Figure 1C). Furthermore, *Mkp-3* expression was also increased by Tg treatment compared with control group (Figure 1D).

Primary mouse hepatocytes were also treated with excess energy to induce chronic ER stress. Results showed that palmitic acid (PA, 50 µM) treatment for four days significantly elevated *Grp78* and *Chop* expression (Figure 1E), and markedly increased *Mkp-3* expression (Figure 1F) compared to control group. These data indicate that long-term, low-dose Tg and PA treatment can effectively induce ER stress in primary mouse hepatocytes, accompanied with promoted hepatic gluconeogenesis and increased *Mkp-3* expression.

### 2.2. Sustained Low-Dose Tg Treatment Induces ER Stress and Promotes Gluconeogenesis and MKP-3 Expression in Mouse Liver

**Figure 2:**
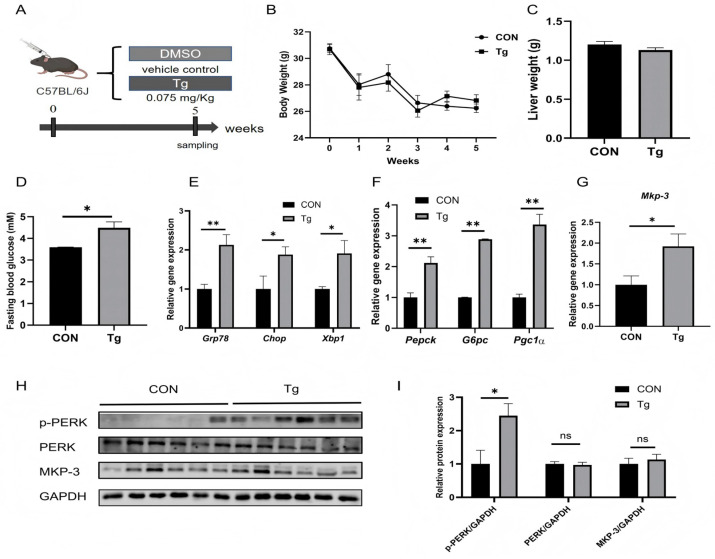
Chronic Tg treatment induced ER stress in mouse liver and promoted gluconeogenesis and *Mkp-3* expression. (**A**) Mice were intraperitoneally injected with Tg (0.075 mg/kg body weight) or equivalent volume of DMSO/saline solution for 35 days. (**B**) Changes in body weight during the treatment (*n* = 6). (**C**) Liver weight (*n* = 6). (**D**) Fasting blood glucose concentrations at harvest time (*n* = 6). (**E**) Expression levels of ER stress marker genes in mouse liver (*n* = 6). (**F**) Expression levels of gluconeogenic genes and their regulator *Pgc1α* in mouse liver (*n* = 6). (**G**) Expression level of the *Mkp-3* gene in mouse liver (*n* = 6). (**H**,**I**) Protein levels of p-PERK and MKP-3 in mouse liver (*n* = 6). Data are presented as mean ± standard error. * *p* < 0.05, ** *p* < 0.01, ns indicates no significant difference.

To investigate the in vivo effects of chronic ER stress mediated by Tg on hepatic gluconeogenesis and *Mkp-3* expression, wild-type mice were intraperitoneally injected with thapsigargin (Tg, 0.075 mg/kg body weight) or equivalent volume of DMSO/saline solution for 35 days (Figure 2A). Results showed that long-term low-dose Tg treatment did not change the body weight or liver weight (Figure 2B,C), but significantly increased fasting blood glucose level compared to the control group (Figure 2D).

Further study revealed that long-term low-dose Tg treatment significantly increased the expression of ER stress marker genes *Grp78*, *Chop* and *Xbp1* compared to the control group (Figure 2E). In addition, hepatic gluconeogenic genes *Pepck* and *G6pc* and their regulator *Pgc1α* were upregulated by chronic Tg treatment compared to the control (Figure 2F), along with increased hepatic *Mkp-3* gene expression (Figure 2G). Given the established role of the PERK pathway in metabolic regulation, the PERK signaling was investigated in the liver. Results showed that the phosphorylation of PERK (p-PERK) was significantly increased by Tg treatment compared to the control, while MKP-3 protein levels were similar between the two groups (Figure 2H,I).

These data indicate that chronic Tg treatment could induce hepatic ER stress and gluconeogenesis, accompanied with hyperglycemia and upregulation of hepatic *Mkp-3* expression.

### 2.3. Excess Energy Intake Induces ER Stress and Promotes Gluconeogenesis and Mkp-3 Expression in the Liver

**Figure 3:**
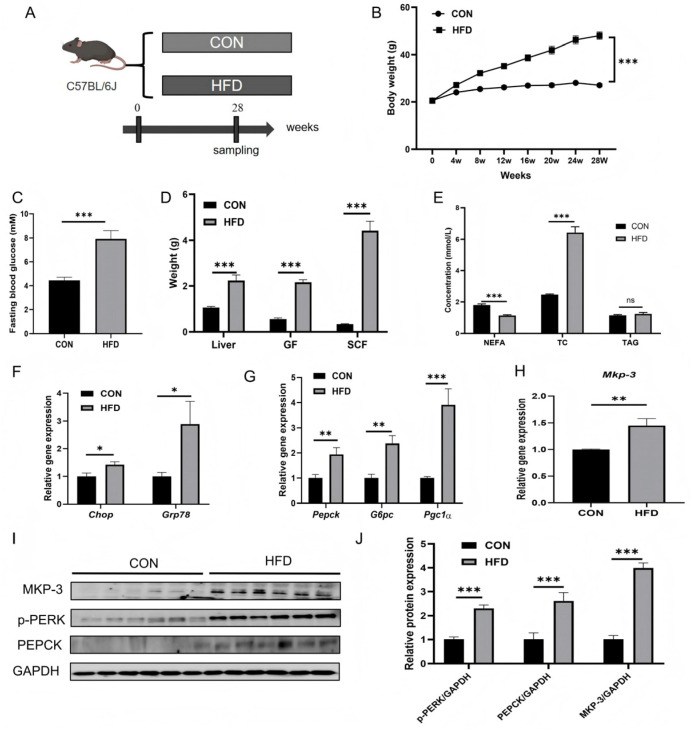
Prolonged excess energy intake induced hepatic ER stress and promoted gluconeogenesis and *Mkp-3* expression. (**A**) Mice were fed with a high-fat diet (HFD) or standard chow diet for 28 weeks. (**B**) Changes in body weight during the treatment (*n* = 12). (**C**) Fasting blood glucose concentration in mice (*n* = 12). (**D**) Weights of liver, gonadal fat (GF) and subcutaneous fat (SCF) in the mice (*n* = 12). (**E**) Serum concentrations of non-esterified fatty acids (NEFA), total cholesterol (TC), and triglyceride (TAG) in the mice (*n* = 12). (**F**) Expression levels of *Grp78* and *Chop* in mouse liver (*n* = 12). (**G**) Expression levels of *Pepck*, *G6pc* and *Pgc1α* in mouse liver (*n* = 12). (**H**) Expression levels of *Mkp-3* in mouse liver (*n* = 12). (**I**,**J**) Protein levels of p-PERK, PERK and MKP-3 in mouse liver (*n* = 6). Data are presented as mean ± standard error. * *p* < 0.05, ** *p* < 0.01, *** *p* < 0.001, ns indicates no significant difference.

To investigate the effects of prolonged excess energy intake on ER stress and gluconeogenesis, mice were fed with a high-fat diet (HFD) for 28 weeks (Figure 3A). Results showed that HFD feeding significantly increased body weight and fasting blood glucose level compared to the control group (Figure 3B,C). In addition, liver weight, gonadal fat (GF) weight, and subcutaneous fat (SCF) weight were markedly higher in HFD- fed mice than those in control lean mice (Figure 3D). Serum total cholesterol (TC) concentration was higher, while that of non-esterified fatty acids (NEFA) was lower in the HFD group compared to the control group, where serum triglyceride (TAG) level remained unchanged (Figure 3E).

Expression of ER stress marker genes *Grp78* and *Chop* (Figure 3F), and gluconeogenic genes *Pepck* and *G6pc,* and *Pgc1a* (Figure 3G) were higher in the liver of HFD- fed mice than those of lean mice. Furthermore, both gene expression and protein levels of MKP-3 were significantly higher in the liver of HFD-fed mice than those of lean mice (Figure 3H–J). In addition, the protein level of p-PERK in the liver was increased by prolonged excess energy intake (Figure 3I,J).

These findings were subsequently validated in swine, a model that is highly homologous to humans (Figure S1A). Similar to results in mice, HFD-fed swine exhibited significantly elevated hepatic expression of ER stress markers *GRP78* and *CHOP* (Figure S1B), and *MKP-3* compared to the control (Figure S1C). These data indicate that prolonged excess energy intake induces ER stress and promotes gluconeogenesis and MKP-3 expression in the liver.

### 2.4. MKP-3 Mediates the Promotion of Hepatic Gluconeogenesis by Chronic ER Stress

**Figure 4:**
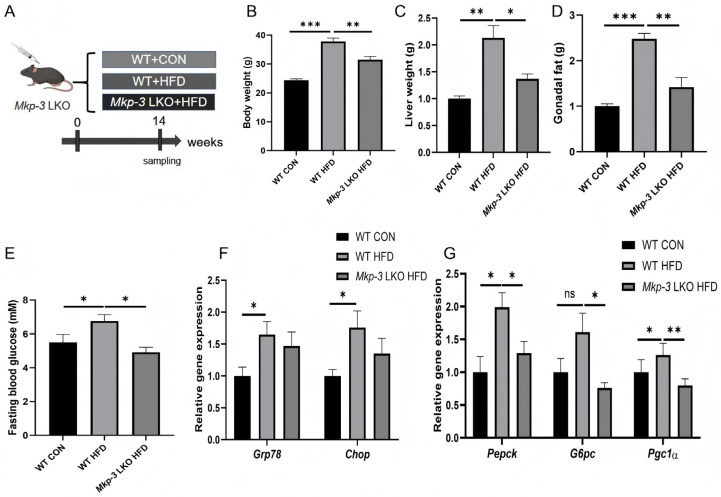
Role of MKP-3 in chronic ER stress promoting hepatic gluconeogenesis. (**A**) Wild-type (WT) and liver-specific *Mkp-3* knockout (*Mkp-3* LKO) mice were fed with a HFD or standard chow diet for 14 weeks. (**B**) Body weight at harvest time (*n* = 5). (**C**) Liver weight (n = 5). (**D**) Gonadal fat weight (*n* = 5). (**E**) Fasting blood glucose concentration (*n* = 5). (**F**) Expression levels of *Grp78* and *Chop* in mouse liver (*n* = 5). (**G**) Expression levels of *Pepck*, *G6pc*, and *Pgc1α* in mouse liver (*n* = 5). Data are presented as mean ± standard error. * *p* < 0.05, ** *p* < 0.01, *** *p* < 0.001, ns indicates no significant difference.

To investigate the role of MKP-3 in ER stress-mediated gluconeogenesis, liver-specific *Mkp-3* knockout (*Mkp-3* LKO) mice and wild-type (WT) mice were fed with a HFD (Figure 4A). Results showed that *Mkp-3* LKO decreased body weight and tissue weight of liver and gonadal fat of those increased by HFD (Figure 4B–D). In addition, compared to the standard chow diet-fed WT mice, HFD-fed WT mice had higher blood glucose level, while this was reversed by *Mkp-3* LKO (Figure 4E).

Further study revealed that the expression of ER stress marker genes *Grp78* and *Chop* was higher in the liver of WT-HFD-fed mice than those in the control group, whereas it was comparable in both WT mice and *Mkp-3* LKO mice under HFD (Figure 4F). Importantly, hepatic gluconeogenic genes *Pepck* and *G6pc*, and their regulator *Pgc1α* were significantly upregulated by prolonged HFD intake, while they were reversed by *Mkp-3* LKO (Figure 4G). These data indicate that MKP-3 could mediate the promotion of hepatic gluconeogenesis by chronic ER stress.

### 2.5. Chronic ER-Stress-Induced MKP-3 Expression Through PERK

**Figure 5:**
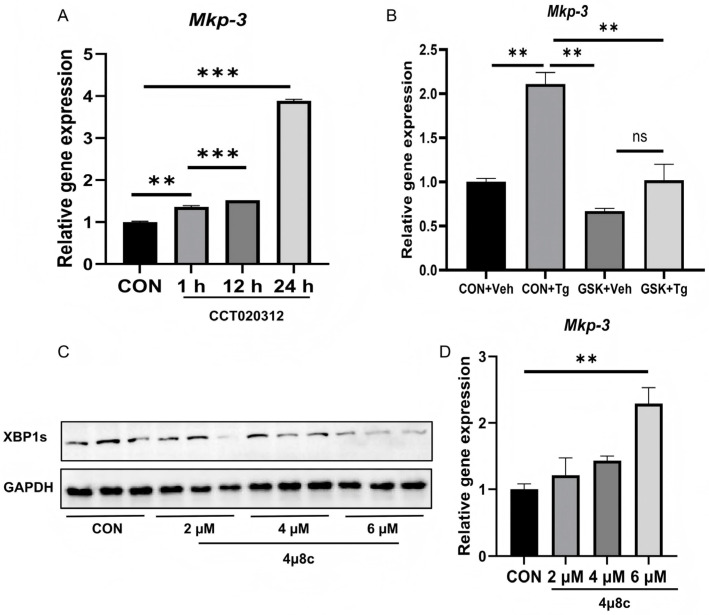
Chronic ER stress promoted *Mkp-3* expression via the PERK pathway. (**A**) Primary mouse hepatocytes were treated with a PERK agonist CCT020312 at 0.4 μM, gene expression level of *Mkp-3* was then measured (*n* = 3). (**B**) Primary mouse hepatocytes were co-treated with Tg and a PERK inhibitor GSK2656157 at 0.04 μM, gene expression level of Mkp-3 was then measured (*n* = 3). (**C**,**D**) Primary mouse hepatocytes were treated with an IRE1 inhibitor 4µ8c, protein level of XBP1 (**C**) and gene expression level of *Mkp-3* were measured (*n* = 3). Data are presented as mean ± standard error. ** *p* < 0.01, *** *p* < 0.001, ns indicates no significant difference.

The molecular mechanism underlying chronic ER stress inducing *Mkp-3* expression was then investigated. Results showed that the PERK agonist CCT020312 significantly increased *Mkp-3* expression compared to the control (Figure 5A). In addition, the PERK inhibitor GSK2656157 blocked the induction effect of chronic Tg treatment on *Mkp-3* expression (Figure 5B). On the other hand, although the IRE1 inhibitor 4µ8c inhibited Xbp1 splicing (Figure 5C), it increased *Mkp-3* expression (Figure 5D). These data indicate that chronic ER stress promotes *Mkp-3* expression primarily through the PERK pathway, but not the IRE1 pathway.

## 3. Discussion

ER stress plays an important role in the regulation of metabolism. Our previous study revealed that acute ER stress might suppress hepatic gluconeogenesis by inducing MKP-3 protein degradation [18]. The current study established chronic ER stress by the administration of an ER stress inducer chemical Tg, or by prolonged excess energy intake in both mouse and swine models. The results indicated that chronic ER stress promoted hepatic gluconeogenesis and *Mkp-3* expression, while liver-specific *Mkp-3* knockout mice (*Mkp-3* LKO) reversed this effect. In addition, chronic ER stress might induce the expression of *Mkp-3* through PERK. Thus, the current study is of significant value for controlling hyperglycemia caused by long-term excess energy intake or chronic chemical-induced liver injury.

Previous report indicates that acute ER stress induced by certain chemicals can reduce hepatic glucose release [18,19]. However, obesity-related ER stress promotes gluconeogenesis [20]. The association between obesity-induced ER stress and insulin resistance is well recognized in metabolic diseases, though the specific molecular mechanisms are not fully understood [21,22]. The findings of the current study support the classical theory of a positive correlation between obesity and chronic ER stress [23]. PA and Tg are two classic inducers for ER stress [23,24]. The current study reveals that prolonged low-dose treatment with Tg or PA could induce chronic ER stress in primary hepatocytes, while promoting gluconeogenesis. Previous studies continuously treat animals with Tg or tunicamycin for 4–6 weeks to induce chronic ER stress in vivo [25,26]. Similarly, the present study treated mice with low-dose Tg for 5 weeks, which induced ER stress and promoted gluconeogenesis. These findings are consistent with the observations in mice that long-term ER stress could be induced by long-term cyclosporin and tunicamycin administration [23,25].

Subsequently, obesity-related chronic ER stress was conducted in mice with a 28-week high-fat feeding, which showed that prolonged high-fat feeding markedly increased expression levels of ER stress marker genes *Grp78* and *Chop* in liver, accompanied with significant increase in p-PERK protein. These findings are consistent with a previous study [23,27]. Furthermore, the study with obese swine yielded similar results.

MKP-3 is a well-established regulator for gluconeogenesis [14,15,16,18,28,29]. MKP-3 stimulates the transcription of gluconeogenic genes PEPCK and G6PC by dephosphorylating FOXO1 [17]. The present study indicated that the expression of *Mkp-3* was increased by chronic ER stress induced by prolonged low-dose chemical treatment or excess energy intake. Further study with *Mkp-3* LKO mice revealed that *Mkp-3* deficiency reversed the increasing of gluconeogenesis by obesity-related chronic ER stress. Taken together, these results and our previous report that MKP-3 mediates acute ER stress to suppress gluconeogenesis [18], suggest that MKP-3 might play important roles in the regulation of gluconeogenesis by ER stress. Previous reports have also demonstrated that MKP-3 is involved in the regulation of glucose and lipids metabolism by dexamethasone, insulin and leptin [14,15,16].

The expression of MKP-3 can be regulated through both transcriptional and post-transcriptional methods. Reports indicated that serum growth factor induced the degradation of MKP-3 through the mTOR pathway [30], leptin decreased MKP-3 protein level through STAT3 [14], and insulin promoted the degradation of MKP-3 protein through the ERK pathway in hepatocytes [15], while acute ER stress stimulated MKP-3 protein degradation without altering its mRNA [18]. On the other hand, gene expression of *MKP-3* can be upregulated by ERK [31]. The present study revealed that chronic ER stress induced *Mkp-3* gene expression. In obese mice, both mRNA and protein levels were increased compared to control lean mice. However, prolonged low-dose Tg administration only increased the mRNA level of *Mkp-3*, but its protein level was unchanged. This might be because long-term chronic ER stress induced gene expression of *Mkp-3*, but acute ER stress suppressed the protein level of MKP-3 as our previous report showed [18]. In the current study, mice were administered with low-dose Tg for 35 days to induce chronic ER stress, and the gene expression of *Mkp-3* was significantly upregulated, supporting the hypothesis that chronic ER stress increases MKP-3 expression. However, liver samples in the current study were harvested 4 h after the last Tg injection. This might induce a short-term acute ER stress, which can decrease the protein level of MKP-3. The protein level of MKP-3 in the long-term low-dose Tg group might be higher than that in the control group 12 h after Tg administration when acute ER stress disappears. However, this will be confirmed in a future study.

IRE1, PERK and ATF6 are classic pathways in ER stress [18,29,32,33]. Among them, the PERK signaling is identified as a regulator for metabolism, particularly hepatic gluconeogenesis, under obesity and insulin resistance status which are accompanied with chronic ER stress [8]. And IRE1 signaling has been reported to suppress gluconeogenesis [34], while the ATF6 pathway is classically associated with the restoration of ER protein folding capacity and cell survival [8], but its role in regulating hepatic glucose metabolism has not been reported. The current study revealed that activation of PERK by its activator CCT020312 upregulated *Mkp-3* expression, while inactivation of PERK by its inhibitor GSK2656157 blocked the increasing effect of chronic Tg administration on the expression of *Mkp-3*. However, the inhibitor of IRE1 (4µ8c) even increased *Mkp-3* expression. These data indicate that chronic ER stress might upregulate *Mkp-3* expression through PERK.

Taken together, the data of the current study and our previous report suggest that ER stress has dual roles in regulating hepatic gluconeogenesis [18], which can be supported by previous reports [35]. In detail, acute ER stress might suppress gluconeogenesis by inducing the degradation of MKP-3 protein, while chronic ER stress promotes gluconeogenesis by promoting *MKP-3* gene expression.

However, there are some limitations in the current study. Firstly, in the mechanism study for chronic ER stress regulating *Mkp-3* expression, only pharmacological inhibitors or activators for PERK and IRE1 were applied. There might be other unknown targets for these pharmacological molecules. *PERK* knock-down or knock-out research will be performed in a future study. Secondly, though the PERK was identified as a mediator for chronic ER stress promoting *Mkp-3* expression, the specific downstream transcription factor that binds to the *Mkp-3* promoter region following PERK activation remains unclear. Thirdly, the validation of ER stress in the current study relied solely on the expression of gene markers (Grp78, Chop, p-PERK and XBP1s), but direct cellular morphological characterization with transmission electron microscopy (TEM) or immunofluorescence microscopy, which provide visual confirmation of ER structural changes [36], was lacking. In future studies, the TEM will be applied to confirm induction of ER stress.

## 4. Materials and Methods

### 4.1. Animal Studies

The animal study protocol was reviewed and approved by the Animal Ethical and Welfare Committee of Sichuan Agricultural University (approve no. 20190122; approval date: 12 March 2019) and was performed in accordance with the National Research Council’s Guide for the Care and Use of Laboratory Animals.

The chronic ER stress in mouse liver was established by prolonged feeding a high-fat diet or long-term administration of a low-dose Tg. For the high-fat diet treatment, twenty-four 4-week-old wild-type mice (C57BL/6N, purchased from Vital River Laboratory Animal Technology Co. Ltd., Beijing, China) with similar body weight were selected and divided into two groups. The control group was fed a standard chow diet, while the other group was fed a high-fat diet (HFD, MD12033, 60% calorie from fat, both diets were purchased from Jiangsu Meidisen Bio-Pharmaceutical Co., Ltd., Taizhou, China) for 28 weeks. Mice were then measured in blood glucose (blood glucose strips were purchased from Beijingyicheng, Beijing, China) and sacrificed for liver harvesting after a 12 h fasting.

For Tg administration, fourteen sixteen-week-old wild-type lean C57BL/6N mice (Vital River Laboratory Animal Technology Co., Ltd., Beijing, China) with comparable body weight were selected and randomly divided into two groups. Mice from the Tg group received 0.075 mg/kg body weight of Tg intraperitoneally for 35 consecutive days, while mice of the control group received equivalent volumes of DMSO/saline (1:100) solvent. Mice were then measured in blood glucose and sacrificed for liver harvesting 4 h after the last administration of Tg, under a 12 h fasting.

The liver-specific *Mkp-3* knockout (Mkp-3 LKO) mice were generated as previously reported [18]. Briefly, *Mkp-3*^loxp/loxp^ mice (S-CKO-13779, Cyagen Biosciences, Guangzhou, China) were mated with albumin-Cre mice (Jackson Laboratory, Bar Harbor, ME, USA) to generate *Mkp-3* LKO (*Mkp-3*^loxp/loxp^, Cre^+^) mice. The *Mkp-3*^loxp/loxp^ littermates were used as the control group. Five male *Mkp-3*^loxp/loxp^ mice were fed with standard chow diet, while another five male *Mkp-3*^loxp/loxp^ mice and five *Mkp-3* LKO mice were fed with the high-fat diet from week of age for 16 weeks. Mice were then measured in blood glucose and sacrificed for liver harvesting after a 12 h fasting.

Twelve sows (Landrace × Large White background) with similar backfat thickness (12.76 ± 13.43 cm) and body weight (233.67 vs. 234.67 kg) were selected and randomly divided into two groups. The control group (n = 6) received a basal diet containing 3% crude fat, while the HFD group (n = 6) received a high-fat diet containing 15% crude fat for 142 d. Sows were then measured with blood glucose, and sacrificed for liver harvesting after a 12 h fasting [37].

Animals were maintained under 12 h light/dark cycle, with a room temperature of 22 ± 2 °C, a humidity of 50–70%, and had free access to water.

### 4.2. Cell Culture and Treatment

Primary mouse hepatocytes were isolated by infusing mouse liver with collagenase as previously reported [38]. Cells were then cultured in high-glucose Dulbecco’s Modified Eagle Medium (DMEM) supplemented with 10% fetal bovine serum (FBS) and 1% penicillin–streptomycin (Gibco, Shanghai, China) at 37 °C in a 5% CO_2_ incubator.

To induce chronic ER stress, cells were treated with the Tg (Sigma, Shanghai, China) at 2 ng/mL or equivalent volume of DMSO for 10 days. For PA treatment, cells were cultured in complete medium supplemented with 50 µM PA (Sigma, Shanghai, China) or NaHCO_3_ for 4 days. Cells were passed every two days with respective fresh medium.

For the IRE1 inhibition assay, cells were incubated in serum-free DMEM supplemented with 4µ8c (Selleck Chemicals, Houston, TX, USA) at 2 µM, 4 µM and 6 µM, or DMSO for 24 h. For the PERK activation assay, cells were incubated in serum-free DMEM for 12 h, followed by treating with 0.4 µM CCT020312 (Selleck Chemicals, Houston, TX, USA) for 1, 12 or 24 h. For PERK inhibition assay, cells were incubated in complete medium supplemented with 2 ng/mL Tg for 8 d, followed by co-treating with Tg and 0.04 µM GSK2656157 (Selleck Chemicals, Houston, TX, USA) for another 2 d. Further details regarding other reagents employed in this study are provided in Table S3.

### 4.3. RNA Extraction and Real-Time PCR

RNA extraction and real-time PCR procedures were performed according to previous report [14]. Briefly, RNA was extracted using Trizol reagent (Sigma, St. Louis, MI, USA) and cDNA was synthesized using a reverse transcription PCR kit (Thermo Fisher Scientific, Shanghai, China). Real-time PCR was performed using a quantitative PCR instrument (7900HT model, ABI Corporation, Carlsbad, CA, USA). Power SYBR Green RT-PCR Reagent (Bio-Rad Laboratories, Hercules, CA, USA) was used and the primer sequences are detailed in Table S1.

### 4.4. Western Blot Analysis

Total proteins were extracted from liver tissue and hepatocytes using a RIPA cell lysis buffer, and the target proteins were detected by Western blotting employing their specific antibodies. Regents and antibody information is provided in Tables S2 and S3.

### 4.5. Statistical Analysis

Data were analyzed using SAS 9.3 software (Cary, NC, USA). Independent *t*-tests were employed to compare differences between groups. Results are presented as mean ± standard error. A *p*-value < 0.05 was considered statistically significant. All cellular studies were repeated at least three times.

## 5. Conclusions

The current study suggests that chronic hepatic ER stress induced by prolonged excess energy intake or chemicals might stimulate gluconeogenesis by upregulating *MKP-3* expression through the PERK pathway.

## Data Availability

The data presented in this study are available on request from the corresponding author.

## Supplementary Materials

The following supporting information can be downloaded at: https://www.mdpi.com/article/10.3390/ijms27062874/s1.
