# Supplementary material for: MAPK Phosphatase-3 Mediates Chronic Endoplasmic Reticulum Stress Promoting Hepatic Gluconeogenesis
Source: Int J Mol Sci. 2026 Mar 22;27(6):2874. doi: 10.3390/ijms27062874 (PMC13026685; doi:10.3390/ijms27062874)
Supplement: Supplementary file 1 [file ijms-27-02874-s001.zip › ijms-4160743-supplementary.pdf]

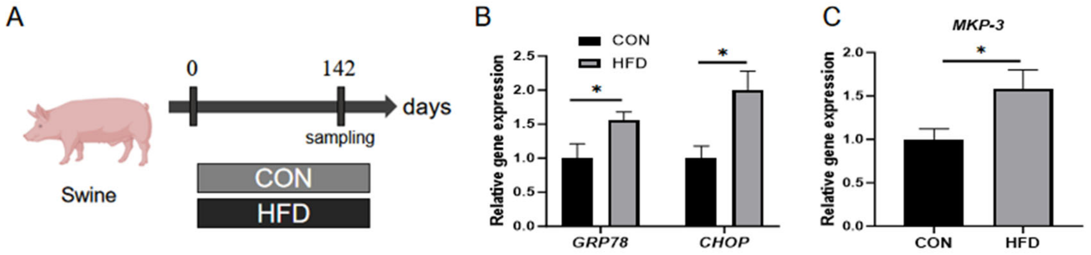

Figure S1. Effects of a high-fat diet on ER stress status and *MKP-3* expression in swine. (A) Swine were fed with a high-fat diet (HFD) or control diet (CON) for 142 days, and then liver samples were collected. (B) Expression levels of the ER stress marker genes *GRP78* and *CHOP* in the liver (n = 6). (C) Expression level of the *MKP-3* gene in the liver (n = 6).

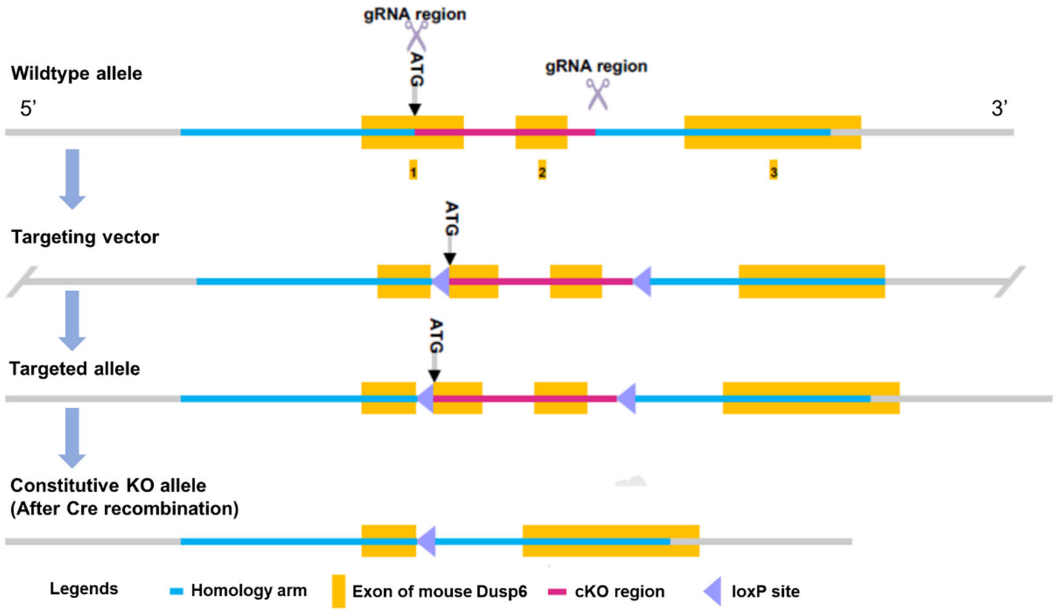

Figure S2. Overview of the strategy to generate the *Mkp-3* LKO mouse.

Table S1. Primers for Real-Time quantitative PCR

Gene	Primer	Sequence (5′→3′)	Accession no.
Mouse			
Grp78	Forward	ATCAGGGCAACCGCATCAC	NM_001163434
	Reverse	TGATGTCCTGCTGCACCGAA	
G6pase	Forward	CGACTCGCTATCTCCAAGTGA	NM_008061
	Reverse	GTTGAACCAGTCTCCGACCA	
Gapdh	Forward	AGGTCGGTGTGAACGGATTTG	NM_008084
	Reverse	TGTAGACCATGTAGTTGAGGTCA	
Pgc1α	Forward	TATGGAGTGACATAGAGTGTGCT	NM_008904
	Reverse	CCACTTCAATCCACCCAGAAAG	
Mkp-3	Forward	TGCGGGCGAGTTCAAATACA	NM_026268
	Reverse	AGCAATGCACCAGGACACCA	
Pepck	Forward	CTGCATAACGGTCTGGACTTC	NM_011044
	Reverse	CAGCAACTGCCCGTACTCC	
Chop	Forward	CACGCACATCCCAAAGCC	NM_007837
	Reverse	GGGCACTGACCACTCTGTT	
Swine			
GAPDH	Forward	TCGGAGTGAACGGATTTGGC	NM_001206359
	Reverse	TGACAAGCTTCCCGTTCTCC	
CHOP	Forward	GCTGGAAAGCAACGCATGAA	NM_001144845.1
	Reverse	ACCATCCGGTCAATCAGAGC	
GRP78	Forward	ATATAAGCGGAGCAGGCGAC	NM_001444629.1
	Reverse	GAGCTCTCACACACACGGAA	
MKP-3	Forward	GGAGTCTGGTAAGCACCTCG	NM_001267842.1
	Reverse	TGCTAATCGCCATCTCCGAC	

Table S2. Antibodies for Western Blot

Antibody	Host	Dilution ratio	Source	Cat. no.
PERK	rabbit	1: 1000	Cell Signaling Technology	3192S
p-PERK	rabbit	1: 1000	Cell Signaling Technology	3179S
PEPCK	rabbit	1: 500	Proteintech	CM04860
G6Pase	rabbit	1: 1000	Bioss	bs-4044R
GAPDH	rabbit	1: 10000	Absin	Abs132994
MKP-3	mouse	1: 500	Santa cruz	sc-377070

Table S3. Reagents

**reagent**	**Source**	**Cat. no.**
RIPA Lysis Buffer	Beyotime	P0013B
PMSF (100mM)	Beyotime	ST506
Color PAGE Gel Rapid Preparation Kit	Epyzime	PG110
iTaq Universal SYBR Green Supermix	Bio-Ard	1725124
4x Laemmli Sample Buffer	Bio-ARD	1610747
DMEM	Gibco	12491015
FBS	Gibco	A5256801
Penicillin-Streptomycin Solution	Beyotime	C0222
